# The Norwegian *PMS2* founder mutation c.989-1G > T shows high penetrance of microsatellite instable cancers with normal immunohistochemistry

**DOI:** 10.1186/1897-4287-12-12

**Published:** 2014-04-21

**Authors:** Eli Marie Grindedal, Harald Aarset, Inga Bjørnevoll, Elin Røyset, Lovise Mæhle, Astrid Stormorken, Cecilie Heramb, Heidi Medvik, Pål Møller, Wenche Sjursen

**Affiliations:** 1 Research Group on Inherited Cancer, Department of Medical Genetics, Oslo University Hospital, Oslo, Norway; 2 Section of Inherited Cancer, Department of Medical Genetics, Oslo University Hospital, Oslo, Norway; 3 Department of Pathology and Medical Genetics, St. Olavs University Hospital, Trondheim, Norway; 4 Department of Laboratory Medicine Children’s and Women’s Health, Norwegian University of Science and Technology, Trondheim, Norway

**Keywords:** Lynch syndrome, PMS2, Immunohistochemistry, Microsatellite instability, Penetrance, Expression

## Abstract

**Background:**

Using immunohistochemistry (IHC) to select cases for mismatch repair (MMR) genetic testing, we failed to identify a large kindred with the deleterious *PMS2* mutation c.989-1G > T. The purpose of the study was to examine the sensitivity of IHC and microsatellite instability-analysis (MSI) to identify carriers of the mutation, and to estimate its penetrance and expressions.

**Methods:**

All carriers and obligate carriers of the mutation were identified. All cancer diagnoses were confirmed. IHC and MSI-analysis were performed on available tumours. Penetrances of cancers included in the Amsterdam and the Bethesda Criteria, for MSI-high tumours and MSI-high and low tumours were calculated by the Kaplan-Meier algorithm.

**Results:**

Probability for co-segregation of the mutation and cancers by chance was 0.000004. Fifty-six carriers or obligate carriers were identified. There was normal staining for PMS2 in 15/18 (83.3%) of tumours included in the AMS1/AMS2/Bethesda criteria. MSI-analysis showed that 15/21 (71.4%) of tumours were MSI-high and 4/21 (19.0%) were MSI-low. Penetrance at 70 years was 30.6% for AMS1 cancers (colorectal cancers), 42.8% for AMS2 cancers, 47.2% for Bethesda cancers, 55.6% for MSI-high and MSI-low cancers and 52.2% for MSI-high cancers.

**Conclusions:**

The mutation met class 5 criteria for pathogenicity. IHC was insensitive in detecting tumours caused by the mutation. Penetrance of cancers that displayed MSI was 56% at 70 years. Besides colorectal cancers, the most frequent expressions were carcinoma of the endometrium and breast in females and stomach and prostate in males.

## Background

Lynch Syndrome (LS) is a multi organ cancer syndrome caused by germline mutations in the four MMR genes *MLH1*, *MSH2*, *MSH6* and *PMS2*. The tumour spectrum includes colorectal- and endometrial cancer as the predominant cancers, and carcinoma of the upper uro-epithelial tract, ovary, hepatobiliary tract, brain, stomach and prostate with lower frequency.

The *PMS2* gene was the last of the four MMR genes to be identified as a cause of LS [1]. It has been suggested that *PMS2* mutations may be associated with a later age of onset of cancer than *MLH1* and *MSH2* mutations [1]. However, reports are few and the exact penetrance and expression of mutations in *PMS2* is considered unknown [2].

Tumours caused by a pathogenic germline mutation in one of the MMR genes have specific characteristics. Firstly, they display microsatellite instability (MSI). Microsatellites are DNA-sequences found throughout the genome where one or units of two or more nucleotides are repeated a certain number of times. A change in the length of these units because of insertion or deletion of repeating units during DNA replication and a failure of the MMR complex to correct these errors is called MSI. Usually, a panel of six markers is used to investigate whether a tumour displays MSI or not. If there is instability in two or more markers the tumour is considered MSI-high. If only one marker shows instability, the tumour is considered MSI-low. Secondly, tumours caused by pathogenic mutations in one of the MMR genes lack expression of the gene product from the mutated gene [3,4]. The presence or absence of gene products from the MMR genes is investigated by immunohistochemistry (IHC) on formaline-fixated tumour specimens. Genetic testing of tumour tissue has several advantages. It is a cheap method that can be used to select patients for more extensive testing of blood by Sanger sequencing and MLPA and one may test deceased relatives.

In general, the sensitivity of MSI and IHC to detect carriers of MMR-mutations is considered to be high [5,6]. However, differences between the genes have been described. It has recently been reported that 95% of cancers occurring in carriers of mutations in *MLH1* or *MSH2* lack staining of gene product from the mutated gene, compared to 71% of cancers in *MSH6* mutation carriers [7]. In addition, whereas 90% of tumours caused by *MLH1* and *MSH2* mutations display MSI [8], tumours caused by *MSH6* mutations show lower levels of MSI and may be classified as MSS or MSI-low [6]. The clinical sensitivity of IHC and MSI to detect carriers of *PMS2*-mutations is not known [9].

It has been demonstrated that *PMS2*-mutation carriers develop cancers that show selective loss of *PMS2* by IHC [1,10,11] and that this is advocated to be followed by *PMS2* mutation testing in blood [1].

In 1999 a large family fulfilling the revised Amsterdam Criteria was referred to the Section for Inherited Cancer at the Oslo University Hospital. IHC was performed on tumours from several of the affected relatives during the following years, showing normal staining of all MMR-genes. Despite the normal staining in tumour tissue, a blood sample from a woman with a prospectively detected endometrial cancer was tested for mutations in all the MMR genes when such testing became available. A pathogenic splice variant *PMS2* c.989-1G > T was detected. The variant had previously been reported [12]. It is now found to be a local frequent mutation in Mid-Norway (unpublished result), and it has also been detected in families living in the south of Norway.

The identification of the mutation in the large family had been delayed several years due to the normal IHC-results in mutation carriers. The aim of the study was to examine the sensitivity of IHC and MSI to identify carriers in cancer cases, and the penetrance and expressions of the mutation.

## Methods

All carriers and obligate carriers of the mutation were identified in the databases at the Section for Inherited Cancer at Oslo University Hospital and at the Department of Medical Genetics at St. Olav’s Hospital in Trondheim. All cancer diagnoses were confirmed from medical files or from The Cancer Registry of Norway.

IHC and MSI-analysis were performed on paraffin blocks from all available tumours. The analyses were performed as described in Sjursen et al. [12]. The PMS2 antibody was monoclonal, clone A16-4, from BD Pharmingen (CA, USA). BD Pharmingen reported to have used recombinant human PMS2 C-terminal half (codon 431–862) as immunogen in antibody development. DNA mutation analyses for the *PMS2* variant c.989-1G > T were performed by Sanger sequencing and the PCR primers and conditions are also described in Sjursen et al. 2009 [12].

As previously described, demonstrated and obligate mutation carriers were included in segregation analysis to determine probability for co-segregation of the mutation and disease by chance [13].

The Kaplan-Meier Algorithm was used to calculate time from birth to any cancer in demonstrated or obligate carriers. Also, penetrances were calculated separately for MSI-high tumours and for tumours that were MSI-high or MSI-low. In the latter analyses cancers with inconclusive results of MSI or no MSI performed were excluded.

No named data was exported from the medical files. All patients alive had at least one genetic counseling session, and all genetic testing included written informed consent and were conducted according to national legislation. The study was approved by the Ethical review board (ref S02030) and the Norwegian Data Inspectorate (ref 2001/2988-2). The present report is one in a series to meet the request from The Norwegian Parliament to report the results of our activities.

## Results

Forty-eight carriers and 8 obligate carriers were identified, giving a total of 56 individuals that were included in the study, 33 women and 23 men. 19/56 (33.9%) were affected with at least one cancer included in the AMS1/AMS2/Bethesda criteria, and 22/56 (39.3%) were affected with any cancer, 13 women and 9 men. Segregation analysis showed 19 informative meioses, giving a probability of co-segregation by chance of (1/2)^*18*^ corresponding with a probability of pathogenicity of 0.999996.

The most frequently observed cancer both in men and women was colorectal cancer, occurring in 12/22 (54.5%) of the affected with a mean age of 46.9 years (range 26–75). In women colorectal-, endometrial- and breast cancer occurred in 6/13 (46.2%), 5/13 (38.5%) and 3/13 (23.1%) respectively. In men, colorectal-,gastric- and prostate cancer, occurred in 6/9 (66.7%), 3/9 (33.3%) and 2/9 (22.2%) respectively (Table 1).

**Table 1:** **Expression of cancers in carriers of *PMS2 *c.989-1G > T**

**Cancer**	**All (*n*** **= 22)**	**Women (*n*** **= 13)**	**Men (*n*** **= 9)**
Colorectum	12 (54.5%)	6 (46.2%)	6 (66.7%)
Stomach	3 (13.6%)	-	3 (33.3%)
Kidney	1 (4.5%)	1 (7.7%)	-
Malignant melanoma	1 (4.5%)	1 (7.7%)	-
Sarcoma	1 (4.5%)	1 (7.7%)	-
Endometrium	-	5 (38.5%)	-
Ovary	-	1 (7.7%)	-
Breast	-	3 (23.1%)	-
Prostate	-	-	2 (22.2%)

**Figure 1:**
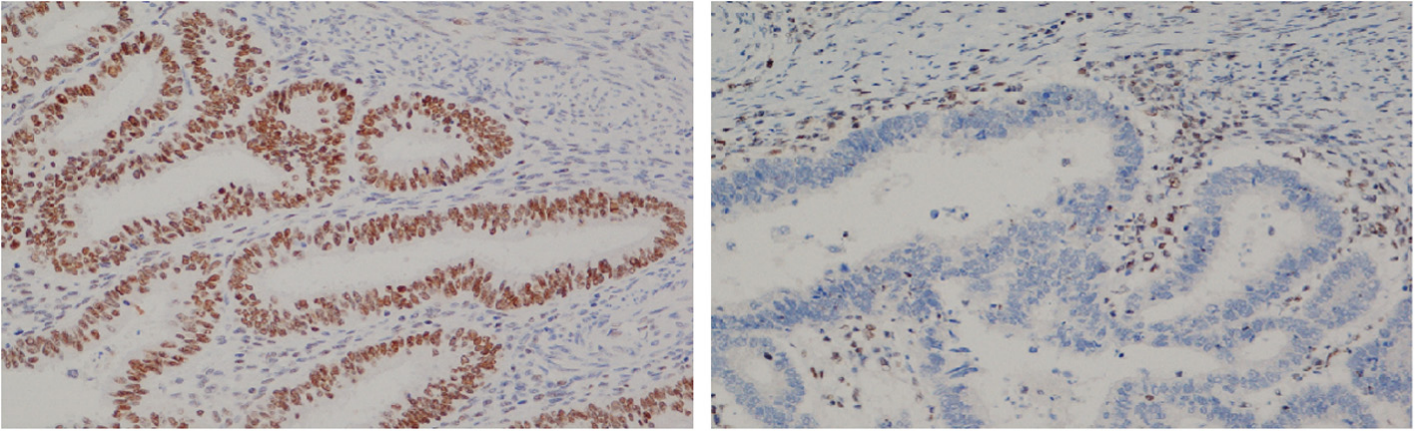
**Endometrial cancers in carriers of *PMS2 *c.989-1G > T with and without expression of PMS2.** IHC analysis of two adenocarcinomas of the endometrium. Immunhistochemical reaction shows expression (left) or no expression (right) of PMS2. Magnification × 20. Lymphocytes in the tumour sample provide positive control. Both tumours displayed MSI.

In sum, IHC was performed on 24 tumours, and 21/24 (87.5%) showed expression of PMS2*.* Of tumours included in AMS1/AMS2/Bethesda criteria 15/18 (83.3%) showed normal staining for PMS2 with IHC. Three breast cancers, one sarcoma, one prostate cancer and one renal cancer, all showed normal IHC for PMS2 (Figure 1).

MSI analysis was performed on altogether 23 tumours in mutation carriers, and 21 gave conclusive results. Nineteen out of twenty-one (90.5%) of the tumours were MSI. Fifteen out of twenty-one (71.4%) were MSI-high and 4/21 (19.0%) of the tumours were MSI-low. The tumours that were MSI-low were one endometrial cancer, one breast cancer, one prostate cancer and one gastric cancer. Six of the tumours available for MSI-analyses were not included in the AMSI/AMSII/Bethesda criteria; three breast cancers, one renal cancer, one prostate cancer and one sarcoma. All but one of the breast cancers and the renal cancer were MSI. The sarcoma and one breast cancer were MSI-high. The prostate cancer and the other breast cancer were MSI-low (Table 2).

**Table 2:** IHC/MSI profile according to type of cancer

**Tumour type**	**IHC performed**	**PMS2 expressed**	**Loss of PMS2 expression**	**MSI performed**	**MSS**	**MSI high**	**MSI low**
Colorectal	11	9	2	9		9	
Gastric	2	2	0	2		1	1
Endometrial	4	3	1	3		2	1
Prostate	1	1	0	1			1
Ovarian	1	1	0	1		1	
Breast	3	3	0	3	1	1	1
Sarcoma	1	1	0	1		1	
Renal	1	1	0	1	1		
Sum	24	21 (87.5%)	3	21	2	15 (71.4%)	4 (19.0%)

The three tumours that showed no staining of PMS2 by IHC were two colorectal cancers and one endometrial cancer. MSI-analysis was performed on one of the colorectal cancers and on the endometrial cancer, and both tumours were MSI-high.

**Figure 2:**
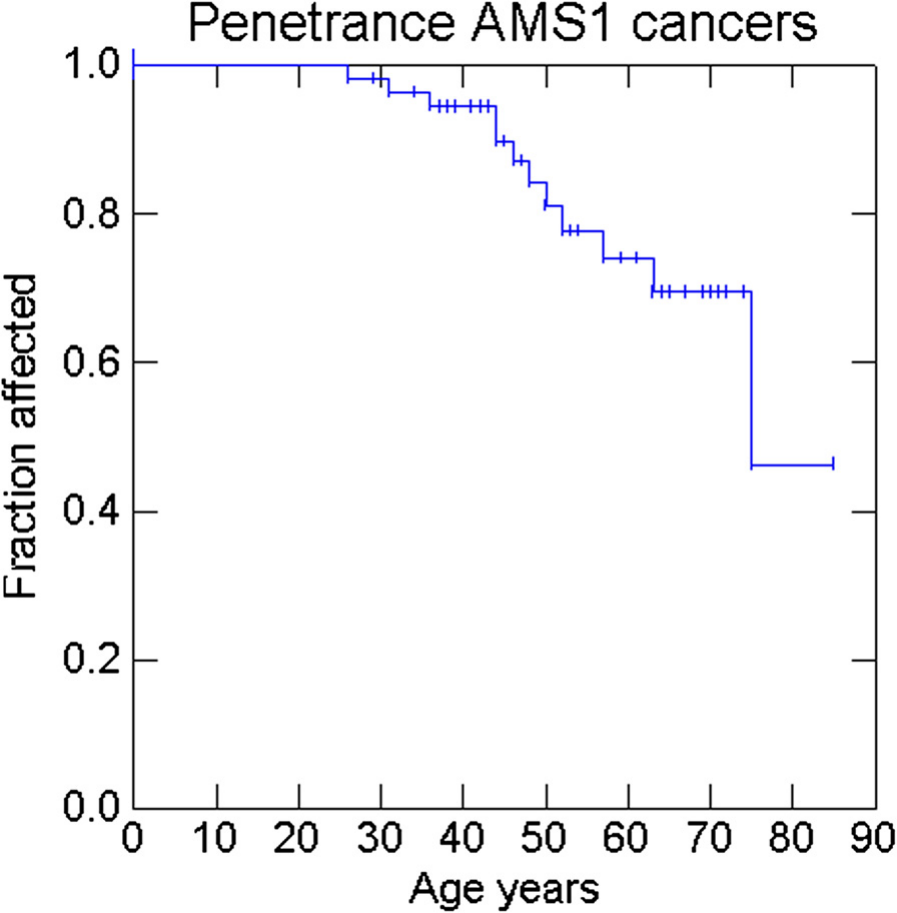
Penetrance of cancers included in the Amsterdam clinical criteria.

**Figure 3:**
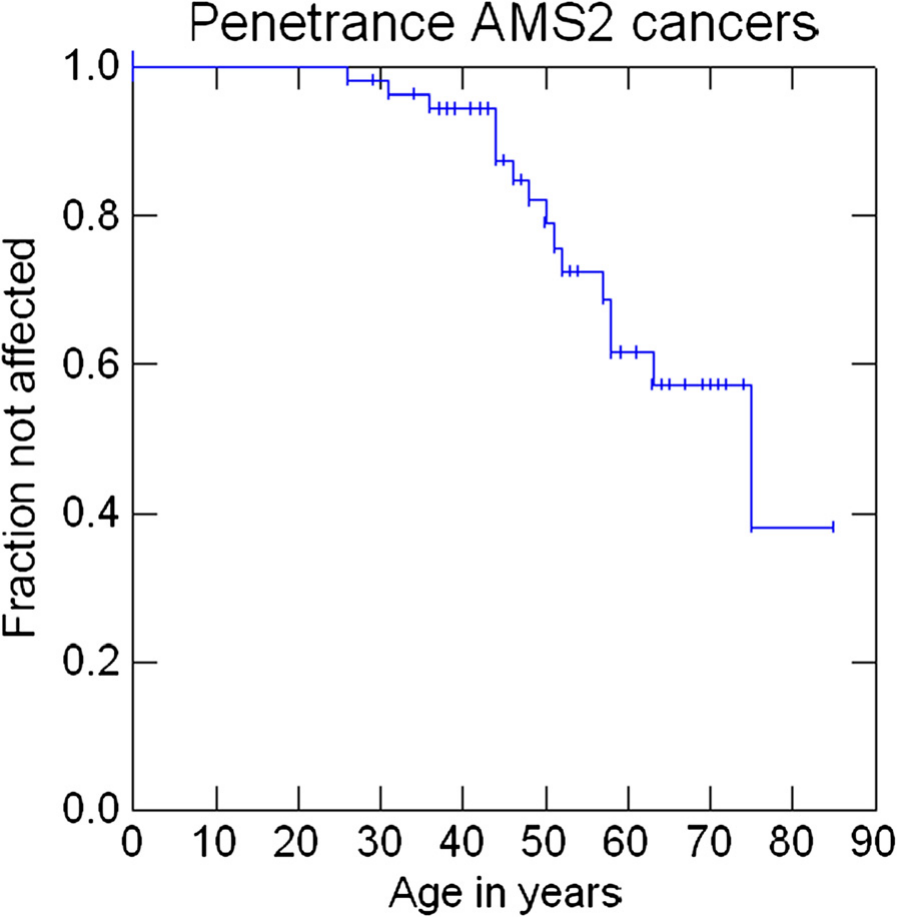
Penetrance of cancers included in the revised Amsterdam clinical criteria.

**Figure 4:**
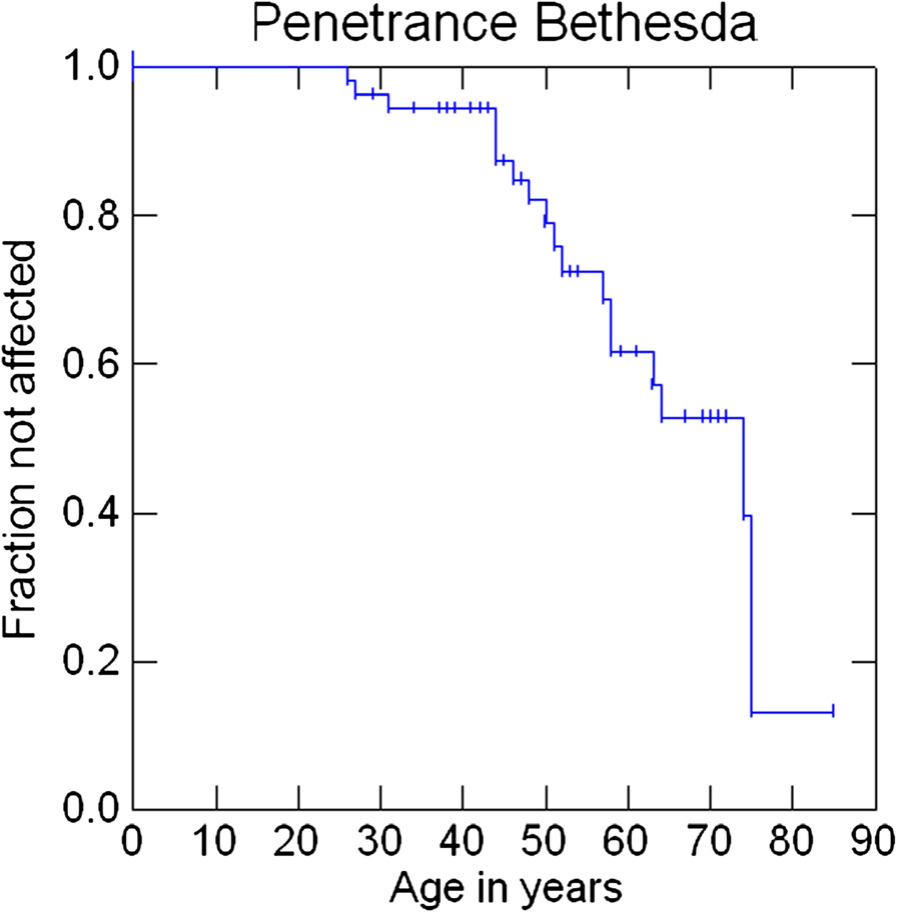
Penetrance of cancers included in the Bethesda clinical criteria.

**Figure 5:**
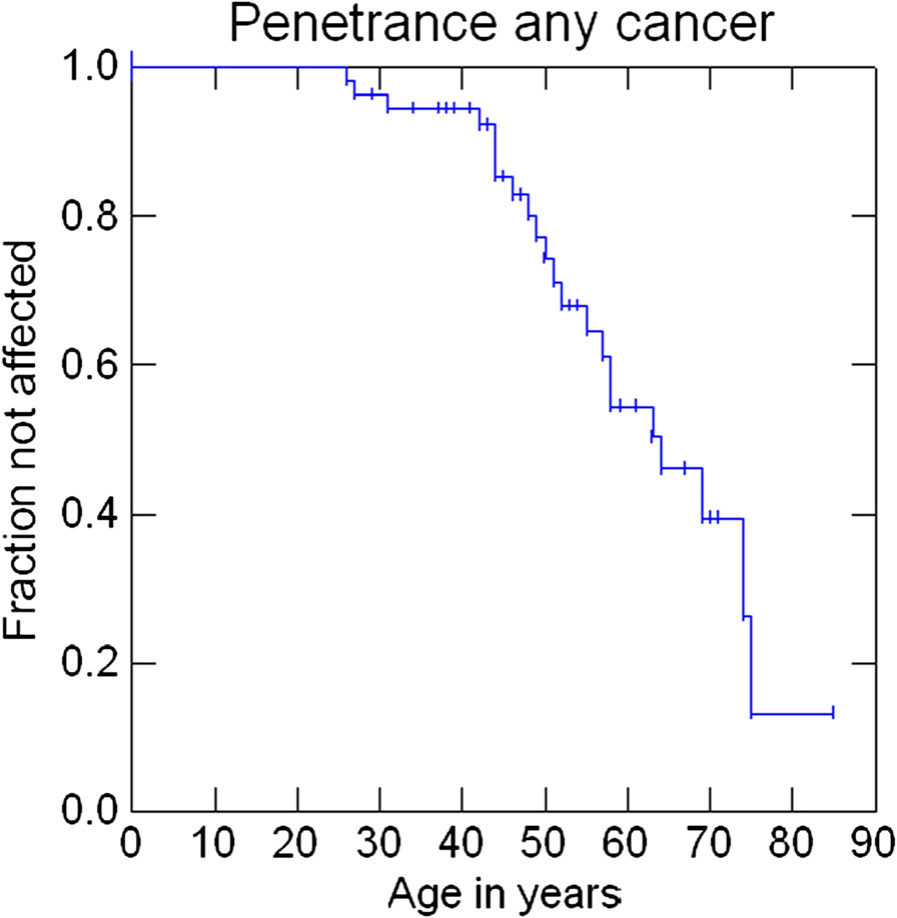
Penetrance of any cancer.

**Figure 6:**
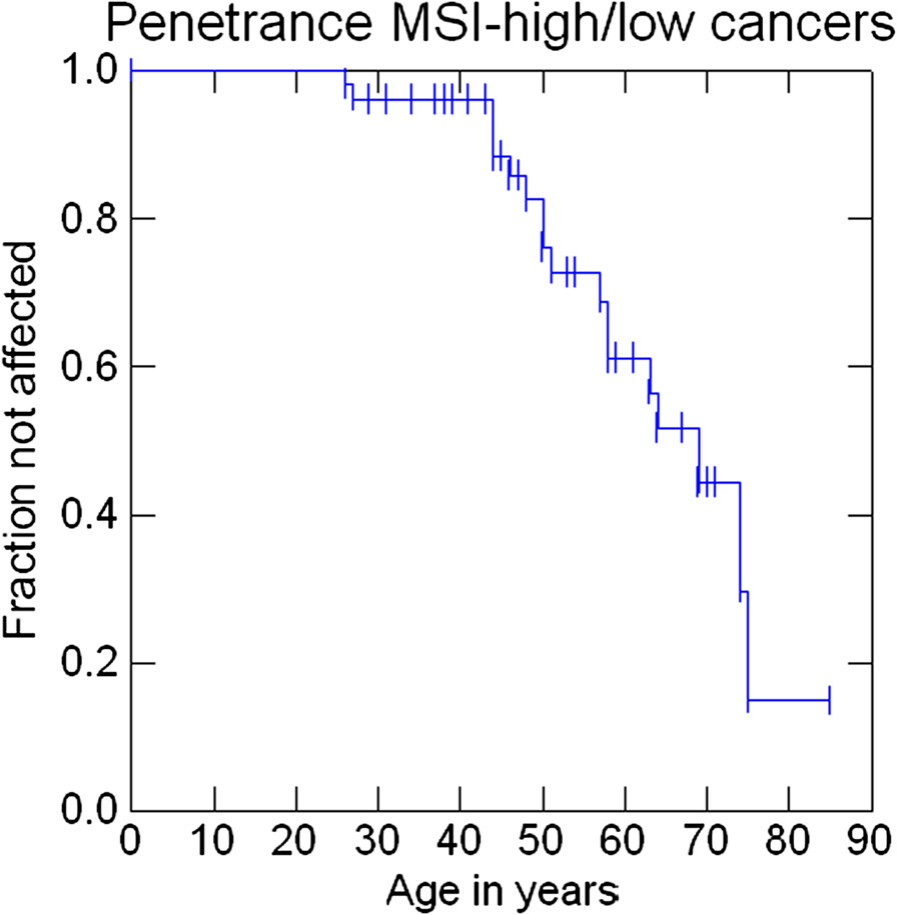
Penetrance of cancers that were either MSI-high or MSI-low.

**Figure 7:**
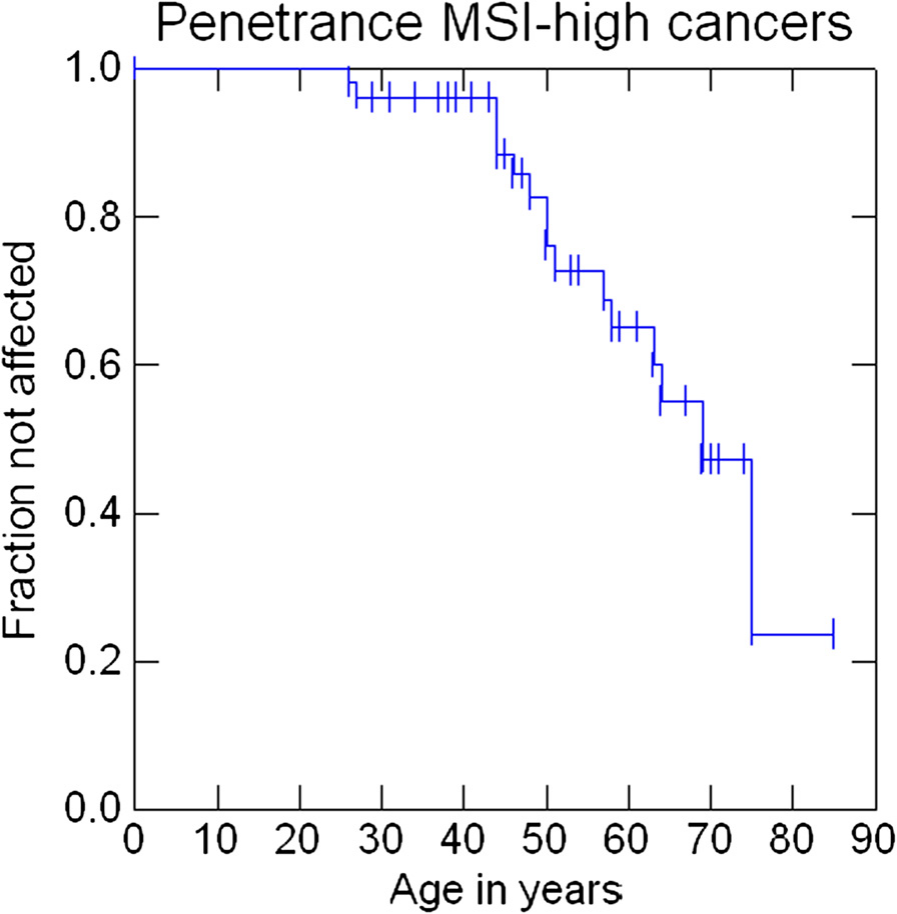
Penetrance of cancers that were MSI-high.

Penetrance at 30, 50 and 70 years was 1.8%, 15.7% and 30.6% for AMS1 cancers (colorectal cancers), 1.8%, 18% and 42.8% for AMS2 cancers, 3.6%, 20.9% and 47.2% for Bethesda cancers and 3.6%, 22.8%, and 60.5% for any cancer. Penetrance of cancers that were either MSI-high or MSI-low at 30, 50 and 70 years was 3.9%, 17.2% and 55.6%. Penetrance of cancers that were MSI-high at 30, 50 and 70 years was 3.9%, 17.2% and 52.2% (Figures 2, 3, 4, 5, 6 and 7).

## Discussion

The splice mutation *PMS2* c.989-1G > T met the InSiGHT criteria class 5 for pathogenicity [14]: mRNA analysis showed that it caused two abnormal transcripts, it co-segregates with disease, more than 2 tumours were MSI-high and has a population frequency <1%. However, whereas 90% of tumours in mutation carriers were MSI-high or MSI-low, only 12.5% of tumours showed abnormal staining of PMS2 by IHC. Penetrance of MSI cancers at age 70 was 56%.

Several studies have demonstrated that *PMS2* mutation carriers can be identified among patients with MSI-high tumours and/or loss of PMS2 in tumour tissue. Gill and colleagues found that 72% of MSI-high tumours, showing normal staining for MLH1, MSH2 and MSH6 showed selective loss of PMS2 [11]. In a series of 1048 unselected consecutive cases of CRC selective loss of PMS2 was observed in 1.5% of tumours, which was similar to the frequency of loss of MSH2. Several mutations in the *PMS2* gene were identified among the patients with PMS2-deficient tumours [10]. Accordingly, tumour testing by IHC and MSI is used as a screening tool to select patients for genetic testing for mutations in *PMS2*. However, the clinical sensitivity of IHC and MSI to detect *PMS2* mutation carriers is not known. In our series, IHC alone would have failed to detect about 85% of the *PMS2*-mutation carriers. We have previously reported a homozygous carrier of *PMS2* c.989-1G > T with Turcot syndrome [12]. cDNA-analysis demonstrated that the splice site mutation leads to two abnormal transcripts, one missing exon 10 and the other missing the first 27 nucleotides of exon 10. The latter may be caused by splicing with a cryptic acceptor site. The first part of exon 10 is highly conserved and it contains the catalytically important 340Lys residue for ATP hydrolysis [15]. It also contains the end of the functional domains DNA mismatch repair protein N-terminal and C-terminal (IPR014763 and IPR013507 respectively). None of the transcripts led to frame shift, and this may prevent them from being degraded by nonsense mediated mRNA decay [16,17]. The normal IHC may therefore reflect that the transcripts are translated into non-functional proteins being transferred to the nucleus where they bind to the epitope of the PMS2 antibody. This hypothesis is supported by the fact that the antibody binds to somewhere between codon 431–862 which is downstream to the deletions in the two gene products of the mutation. Further the PMS2 exon 10 deletions in the transcripts do neither affect the MLH1 binding domain (codon 675–850 [18]) nor the nuclear localisation signal (codon 625–632 [19]). Thus, the low sensitivity of IHC to detect tumours caused by the *PMS2* splice mutation is in keeping with a non-functional protein located at the right place in the cell detectable by IHC. Interestingly, a recent study on discrepancies between IHC and MSI results reported that of 646 consecutive tumours investigated, as many as 11.8% of the tumours that were MSI high showed normal expression of all MMR genes. Eight of these 12 tumours were from patients that had a first degree relative with a LS associated cancer. It is not known how many of the patients with MSI-high tumours that carried a MMR mutation [20].

We observed that all colorectal-, gastric, endometrial-, prostate- and ovarian cancers and 2/3 of breast cancers occurring in carriers of the *PMS2* mutation displayed MSI. In addition, the sarcoma was also MSI high. Of the tumours investigated, only the renal cancer and one breast cancer were MSS. MSI analysis is considered a sensitive method to identify MMR mutation carriers, and one could hypothesize that all cancers caused by a pathogenic germline MMR mutation would display MSI. However, it is well known that tumours caused by *MSH6* mutations may show lower levels of MSI and may be classified as MSS or MSI-low [6]. The fraction of tumours that display MSI may also be different for different cancer types. Gylling and colleagues reported that 100% of gastric cancers and more than 90% of colorectal cancers in MMR mutation carriers (>90% were carriers of *MLH1* mutations) were MSI high compared to 63% of endometrial cancers and 25% of renal cancers [21]. Similarly, it has been reported that 60% and 45% of breast cancers occurring in *MSH2* and *MLH1* mutation carriers respectively were MSI high, whereas all breast cancers in *MSH6* mutation carriers were MSS [22]. Our observations indicate that MSI analysis may be used to identify *PMS2* mutation carriers among patients with extra-colonic as well as colonic cancers. However, larger numbers are required to assess whether the fraction of colonic and extra-colonic tumours that display MSI are different from the other MMR genes.

A recent study has demonstrated that IHC has a low sensitivity in identifying pathogenic *MSH6*-mutations, as almost 24% of adenocarcinomas in carriers of pathogenic *MSH6* mutations showed normal staining of this gene [23]. The explanation for this finding remains unknown. We observed that in carriers of *PMS2* c.989-1G > T 83% of cancers included in the AMS1/AMS2/Bethesda clinical criteria had normal expression of PMS2. Whether the sensitivity of IHC to identify carriers of *PMS2*-mutations is similar to *MSH6* or to the high sensitivity reported for tumours caused by *MLH1*- or *MSH2*-mutations cannot be addressed in the present study where carriers of only one mutation were included, but needs further clarification.

The exact penetrance and expression associated with mutations in *PMS2* is not known [2]. Previous reports have referred to the gene as giving an attenuated form of Lynch Syndrome, a lower risk of CRC and a later age of onset than what has been described for *MLH1* and *MSH2*[1]. Several studies have also demonstrated that the majority of families do not fulfill the clinical criteria for LS [1,10,11,24,25]. We observed that penetrance at 70 years for CRC was 30% and 56% for MSI cancers. This may be in keeping with previous studies, and may indicate that risk of CRC is lower than what has been reported for carriers of mutations in *MLH1* and *MSH2*[26]. We also observed that after colorectal cancer, endometrial- and breast cancer were the most commonly observed cancers in women, and gastric- and prostate cancers in men.

## Conclusions

The class 5 pathogenic splice mutation *PMS2* c.989-1G > T had a penetrance for MSI cancers of 56% at 70 years of age. Colorectal cancer was most frequently seen, while expressions included breast-, gastric- and prostate cancers as well. IHC detected only 12.5% of the tumours caused by the mutation. Our observations illustrate the importance of performing both IHC and MSI analysis when selecting patients for MMR genetic testing.

## Abbreviations

AMS1: The original Amsterdam clinical criteria; AMS2: The revised Amsterdam clinical criteria; Bethesda: The Bethesda clinical criteria; IHC: Immunohistochemistry; LS: Lynch Syndrome; MMR: Mismatch repair; MSI: Microsatellite instability.

## Competing interests

The authors declare that they have no competing interests.

## Authors’ contributions

EMG took part in the genetic work up of the families including genetic counseling, contributed to the design of the study, carried out the compilation of the data, the statistical analysis, and coordinated formatting and writing of the manuscript. HA performed the IHC tumour testing. IB took part in the genetic work up of the families including genetic counseling and compiled the data from St. Olavs Hospital. ER performed the IHC tumour testing. LM was involved in the genetic work up of the families including genetic counseling, conceived the study and was involved in its design and formatting of the manuscript. AS was involved in the genetic work up of the families including genetic counseling, was involved in the study design, and formatting of the manuscript. CH was involved in the genetic work up of the families and contributed to the formatting of the manuscript. HM was involved in the genetic work up of the families including genetic counseling, and contributed to the formatting of the manuscript. PM was involved in the genetic work up of the families, including the genetic counseling, conceived the study, contributed to the design of the study, the statistical analysis and the formatting and writing of the manuscript. WS performed the molecular genetic testing, conceived the study and was involved in its design and formatting of the manuscript. All authors read and approved the final manuscript.
